# Supplementary material for: Resolving multisensory and attentional influences across cortical depth in sensory cortices
Source: eLife. 2020 Jan 8;9:e46856. doi: 10.7554/eLife.46856 (PMC6984812; doi:10.7554/eLife.46856)
Supplement: Supplementary file 1. — Notes: Percentage of target responses (mean and STD across subjects) in the six conditions of our 2 × 3 experimental design. n = 11 Please note that responses to visual targets under auditory attention and auditory targets under visual attention are false alarms. [file elife-46856-supp1.docx]

|  | Stimuli |  | | Auditory | | | | | |  | | Visual | | | | |  | | Audio-visual | | | |
| --- | --- | --- | --- | --- | --- | --- | --- | --- | --- | --- | --- | --- | --- | --- | --- | --- | --- | --- | --- | --- | --- | --- |
| Targets |  |  | |  |  |  |  |  |  |  | |  |  |  |  |  |  | |  |  |  |  |
|  |  |  | | Attend to auditory | | | | | | | | | | | | | | | | | | |
| Auditory | | |  | | 80.11% | ( | 13.92% | ) |  | | 93.18% | | ( | 7.63% | ) |  | | 67.05% | | ( | 15.08% | ) |
| Visual | | |  | | 6.82% | ( | 7.63% | ) |  | | 2.27% | | ( | 4.21% | ) |  | | 11.36% | | ( | 7.82% | ) |
|  |  |  | | Attend to visual | | | | | | | | | | | | | | | | | | |
| Auditory | | |  | | 4.55% | ( | 11.21% | ) |  | | 6.25% | | ( | 6.25% | ) |  | | 7.95% | | ( | 7.95% | ) |
| Visual | | |  | | 65.91% | ( | 16.14% | ) |  | | 69.32% | | ( | 14.91% | ) |  | | 65.91% | | ( | 11.98% | ) |
