# Supplementary material for: Resolving multisensory and attentional influences across cortical depth in sensory cortices
Source: eLife. 2020 Jan 8;9:e46856. doi: 10.7554/eLife.46856 (PMC6984812; doi:10.7554/eLife.46856)
Supplement: Supplementary file 2. — Notes: ‘Number of vertices’ refers to the vertices with valid data at all the sampled cortical depths. This vertex count was divided by the total number of vertices included in the initial ROI definition to compute the ‘Fraction of the ROI covered’. Note that those numbers are pooled over both hemispheres. n = 11 [file elife-46856-supp2.docx]

|  | **Number of vertices (*10^3)** | | | | | | |  | | | **Proportion of ROI covered** | | | | | | | | | | |
| --- | --- | --- | --- | --- | --- | --- | --- | --- | --- | --- | --- | --- | --- | --- | --- | --- | --- | --- | --- | --- | --- |
| **ROI** | **mean** | **STD** | **range** | | | | | | |  | | | **mean** | | **STD** | | **range** | | | | |
| **A1** | 19.64 | 3.60 | ( | 15.05 | - | 24.30 | ) | |  | | | 0.96 | | 0.06 | | ( | | 0.84 | - | 1.00 | ) |
| **PT** | 10.17 | 2.31 | ( | 6.85 | - | 14.36 | ) | |  | | | 0.96 | | 0.06 | | ( | | 0.79 | - | 1.00 | ) |
| **V1** | 34.54 | 6.54 | ( | 24.64 | - | 48.44 | ) | |  | | | 0.97 | | 0.02 | | ( | | 0.94 | - | 1.00 | ) |
| **V2-3** | 68.74 | 12.05 | ( | 43.59 | - | 87.51 | ) | |  | | | 0.82 | | 0.08 | | ( | | 0.64 | - | 0.90 | ) |
