# Supplementary material for: Resolving multisensory and attentional influences across cortical depth in sensory cortices
Source: eLife. 2020 Jan 8;9:e46856. doi: 10.7554/eLife.46856 (PMC6984812; doi:10.7554/eLife.46856)
Supplement: Supplementary file 3. — Using 2 (shape parameter: constant, linear) x 2 (ROI: primary, non-primary) linear mixed effects models, we performed the following statistical comparisons in a 'step down procedure': [file elife-46856-supp3.docx]

|  |  | **linear or constant** | |  |  | **constant** | |  | | **linear** | | |
| --- | --- | --- | --- | --- | --- | --- | --- | --- | --- | --- | --- | --- |
| **[A-fix]Att_A, Att_V** | **mean(A1, PT)** | F(2,40)=136.799 | **p<0.001** |  |  | t(10)=11.537 | **p<0.001** | | a | | F(1,20)=52.749 | **p<0.001** |
|  |  |  |  |  | **A1** | t(10)=11.906 | **p<0.001** | | a | | t(10)=8.152 | **p<0.001** |
|  |  |  |  |  | **PT** | t(10)=7.284 | **p<0.001** | | a | | t(10)=10.761 | **p<0.001** |
|  |  |  |  |  |  |  |  | |  | |  |  |
| **[V-fix]Att_A, Att_V** | **mean(V1, V23)** | F(2,40)=158.364 | **p<0.001** |  |  | t(10)=9.864 | **p<0.001** | | a | | F(1,20)=49.620 | p<0.001 |
|  |  |  |  |  | **V1** | t(10)=8.152 | **p<0.001** | | a | | t(10)=6.154 | **p<0.001** |
|  |  |  |  |  | **V2-3** | t(10)=10.761 | **p<0.001** | | a | | t(10)=7.675 | **p<0.001** |
