# Supplementary material for: Resolving multisensory and attentional influences across cortical depth in sensory cortices
Source: eLife. 2020 Jan 8;9:e46856. doi: 10.7554/eLife.46856 (PMC6984812; doi:10.7554/eLife.46856)
Supplement: Supplementary file 4. — Using 2 (shape parameter: constant, linear) x 2 (ROI: primary, non-primary) linear mixed effects models, we performed the following statistical comparisons in a 'step down procedure': [file elife-46856-supp4.docx]

| **A) BOLD profile** |  |  |  |  | |  | |  | |  |  | |  | |  |
| --- | --- | --- | --- | --- | --- | --- | --- | --- | --- | --- | --- | --- | --- | --- | --- |
|  |  | **linear or constant** | | |  | |  | | **constant** | | |  | | **linear** | |
| **[AV - V]Att_A, Att_V** | **mean(V1, V23)** | F(2,40)=0.723 | p=0.491 |  | |  | |  | |  |  | |  | |  |
|  |  |  |  |  | |  | |  | |  |  | |  | |  |
|  |  |  |  |  | |  | |  | |  |  | |  | |  |
| **B) Decoding profile** |  |  |  |  | |  | |  | |  |  | |  | |  |
|  |  | **linear or constant** | | |  | |  | | **constant** | | |  | | **linear** | |
| **[AV VS V]att A, att V** | **mean(V1, V23)** | F(2,40)=0.696 | p=0.505 |  | |  | |  | |  |  | |  | |  |
