# Supplementary material for: Resolving multisensory and attentional influences across cortical depth in sensory cortices
Source: eLife. 2020 Jan 8;9:e46856. doi: 10.7554/eLife.46856 (PMC6984812; doi:10.7554/eLife.46856)
Supplement: Transparent reporting form [file elife-46856-transrepform.docx]

***eLife’s* transparent reporting form**

We encourage authors to provide detailed information *within their submission* to facilitate the interpretation and replication of experiments. Authors can upload supporting documentation to indicate the use of appropriate reporting guidelines for health-related research (see [EQUATOR Network](http://www.equator-network.org/%20)), life science research (see the [BioSharing Information Resource](https://biosharing.org/)), or the [ARRIVE guidelines](http://www.plosbiology.org/article/info:doi/10.1371/journal.pbio.1000412) for reporting work involving animal research. Where applicable, authors should refer to any relevant reporting standards documents in this form.

If you have any questions, please consult our Journal Policies and/or contact us: [editorial@elifesciences.org](mailto:editorial@elifesciences.org).

**Sample-size estimation**

- You should state whether an appropriate sample size was computed when the study was being designed
- You should state the statistical method of sample size computation and any required assumptions
- If no explicit power analysis was used, you should describe how you decided what sample (replicate) size (number) to use

Please outline where this information can be found within the submission (e.g., sections or figure legends), or explain why this information doesn’t apply to your submission:

Because laminar analyses on human fMRI data is a new emerging field, no studies were available that we could use to compute a formal power analysis for our comparisons of interest. Because submillimeter resolution fMRI maximizes the insights gained at the subject-specific level, we acquired 4 fMRI runs in two days, i.e. 302 fMRI scans per subject to maximize the reliability with which estimated BOLD-response within each subject. Previous studies that used laminar analyses in fMRI included 3 – 21 subjects. Given this range of number subjects and scans for each subject, we then decided to obtain 13 number of subjects.

**Replicates**

- You should report how often each experiment was performed
- You should include a definition of biological versus technical replication
- The data obtained should be provided and sufficient information should be provided to indicate the number of independent biological and/or technical replicates
- If you encountered any outliers, you should describe how these were handled
- Criteria for exclusion/inclusion of data should be clearly stated
- High-throughput sequence data should be uploaded before submission, with a private link for reviewers provided (these are available from both GEO and ArrayExpress)

Please outline where this information can be found within the submission (e.g., sections or figure legends), or explain why this information doesn’t apply to your submission:

From the methods section: “Each fMRI run included 18 blocks (3 blocks for each condition in our 2 X 3 design) and lasted 15 minutes. There were 4 runs per participant yielding 12 blocks per condition per participant.”

2 participants were excluded because they had not completed all the MRI sessions.

**Statistical reporting**

- Statistical analysis methods should be described and justified
- Raw data should be presented in figures whenever informative to do so (typically when N per group is less than 10)
- For each experiment, you should identify the statistical tests used, exact values of N, definitions of center, methods of multiple test correction, and dispersion and precision measures (e.g., mean, median, SD, SEM, confidence intervals; and, for the major substantive results, a measure of effect size (e.g., Pearson's r, Cohen's d)
- Report exact p-values wherever possible alongside the summary statistics and 95% confidence intervals. These should be reported for all key questions and not only when the p-value is less than 0.05.

Please outline where this information can be found within the submission (e.g., sections or figure legends), or explain why this information doesn’t apply to your submission:

Individual data are presented as scatters on the violin plots of the figures. A supplementary figure includes surface projections of several subjects.

Unless mentioned otherwise we report mean and SEM.

For neuroimaging results :

All statistical inferences were made using exact permutation tests. The bias-corrected and accelerated bootstrap 95% confidence interval of the effect size (as Cohen’s d) are reported where appropriate. Given our sample size we used Hedges and Olkin’s method to correct for bias in our effect size estimation.

For statistical inference on the behavioural results we used repeated measures ANOVA (results were corrected for non-sphericity using Greenhouse-Geiser correction).

(For large datasets, or papers with a very large number of statistical tests, you may upload a single table file with tests, Ns, etc., with reference to sections in the manuscript.)

**Group allocation**

- Indicate how samples were allocated into experimental groups (in the case of clinical studies, please specify allocation to treatment method); if randomization was used, please also state if restricted randomization was applied
- Indicate if masking was used during group allocation, data collection and/or data analysis

Please outline where this information can be found within the submission (e.g., sections or figure legends), or explain why this information doesn’t apply to your submission:

This information does not apply here as this was a within subject design.

**Additional data files (“source data”)**

- We encourage you to upload relevant additional data files, such as numerical data that are represented as a graph in a figure, or as a summary table
- Where provided, these should be in the most useful format, and they can be uploaded as “Source data” files linked to a main figure or table
- Include model definition files including the full list of parameters used
- Include code used for data analysis (e.g., R, MatLab)
- Avoid stating that data files are “available upon request”

Please indicate the figures or tables for which source data files have been provided:

The raw data of the results presented here are available in a BIDS format upon request.

Derivative data (sufficient to recreate figures 2A, 3A and 4A) are available on the OSF project of this study: https://osf.io/7ka5j/?view_only=eb7a6fcb5be74bb891d62c6b0e81dabf

The code for the analysis of this project is available here:

https://github.com/Remi-Gau/AV-Attention-7T_code
